# Editorial: Recent advances in vascular neurosurgery

**DOI:** 10.3389/fsurg.2023.1159237

**Published:** 2023-04-03

**Authors:** Ilgiz Gareev, Ozal Beylerli, Albert Sufianov, Philipp Taussky

**Affiliations:** ^1^ Educational and Scientific Institute of Neurosurgery, Рeoples’ Friendship University of Russia (RUDN University), Moscow, Russia; ^2^ Department of Neurosurgery, Sechenov First Moscow State Medical University (Sechenov University), Moscow, Russia; ^3^ Beth Israel Deaconess Medical Center, Harvard Medical School Boston, Boston, MA, United States

**Keywords:** endovascular therapy, cerebrovascular disease, vascular neurosurgery, minimally invasive, advances

**Editorial on the Research Topic** Recent advances in vascular neurosurgery

Vascular pathology is one of the most complex branches of neurosurgery. The most common diseases for which neurosurgical treatment is performed are cerebral aneurysms (ruptured and un-ruptured), arteriovenous malformations (AVMs), carotid cavernous fistulas (CCF), brachiocephalic artery stenosis (narrowing of the vessels supplying the brain) (1). Like no other field of medicine, vascular neurosurgery has come a long way in recent years thanks to progress in the field of new methods of minimally invasive interventions. In order to best understand the latest advances in vascular neurosurgery, it is necessary to evaluate the possibilities of endovascular surgical techniques. One of the fastest growing and modern areas in vascular neurosurgery is endovascular neurosurgery. Endovascular neurosurgery allows for the most complex surgical interventions (2). The main advantage of endovascular interventions is low trauma, fast postoperative rehabilitation of patients, accessibility for patients with severe concomitant diseases. With the knowledge that we have and will continue to acquire, it becomes clear that the endovascular intervention technique can be a first-line therapy, as in the case of ruptured or giant cerebral aneurysms (3). Or the endovascular intervention technique may be a second-line therapy after conservative therapy, as in the case of cerebral atherosclerosis (3).

The successes of endovascular neurosurgery over the past thirty years are based on the emergence of new polymers and metal alloys, on the rapid introduction of electronics into medicine, on the development of unprecedented implantable devices. Endovascular neurosurgery has evolved along with improvements in endovascular materials and improved techniques for applying these materials to the target. At the same time, the introduction of new materials led to improved treatment results (4). For example, a detachable coil system has been replaced by a removable balloon in the treatment of cerebral aneurysms, and liquid non-adhesive viscous embolic agents (Onyx) and n-butylcyanoacrylate (n-BCA) are routinely used in the treatment of cerebral arteriovenous malformation. Similarly, improved outcomes have encouraged neurosurgeons to expand indications for endovascular neurosurgery from proximal-distal arteries and from simple to complex lesions (4, 5).

In addition, endovascular therapy can complement open surgery, as in the case of carotid disease. Combined use of endovascular technique with open surgery, as in the case of tumor resections, AVM treatments or complex aneurysms, has become common (6). Basilar artery aneurysms or AVM are the most difficult to access and technically difficult for microsurgical intervention. Endovascular treatment of aneurysms of this localization is a priority, due to the following factors: (1) a higher incidence of complications of microsurgical treatment of basilar artery aneurysms compared to aneurysms of the anterior circulation of the brain; (2) the development of severe complications due to the proximity of perforating arteries that feed the brain stem structures, as well as cranial nerves; (3) limitations of surgical approaches to achieve sufficient visualization of the vascular anatomy in this deep area and limited ability to provide adequate space to control the proximal part of the artery; (4) greater safety of endovascular interventions; (5) endovascular technologies make it possible to treat AVMs rarely in curative fashion but more commonly to allow safer surgery or radiosurgery.

Endovascular modalities also have limitations mostly relating to its durability: Coiled aneurysms show a higher recurrence rates than clipped aneurysms and we see a higher number of incompletely treated aneurysms and AVMs after endovascular treatment. However, as technology advances, so will the applications of endovascular techniques, and the boundaries between medical disciplines such as neurology, neurosurgery, and radiology will continue to blur. For these reasons, neurosurgery remains the ideal discipline for understanding when and how to use endovascular techniques to treat all cerebrovascular diseases. For instance, even before the early 2000s, ischemic stroke was a non-surgical disease in which the best medical treatment with systemic intravenous thrombolysis was only marginally effective. However, the persistence of vascular surgeons in the surgical treatment of mechanical obstruction of large vessel occlusion (LVO) has led to one of the most profound paradigm shifts in modern medicine and neurosurgery in particular (7).

Unfortunately, within the framework of one editorial article it is impossible to cover all areas of development of the still young specialty—endovascular neurosurgery. Nevertheless, only the specialist who has mastered the management of modern angiographic complexes and clearly understands their capabilities, programs and control functions will be able to know, be able to and master the methods of endovascular surgery. Another important rule for the safe and effective performance of endovascular operations is to correctly navigate the instruments—catheters, guidewires, sheaths, implantable devices, stents and embolisms. Endovascular treatment methods are actively introduced into neurosurgery. These are high-tech, minimally invasive, organ-preserving technologies, which are a system of methods for the refined diagnosis and treatment of cerebrovascular diseases, and in some cases, provide an alternative to traditional surgical interventions.
